# Determinants of image quality of rotational angiography for on-line assessment of frame geometry after transcatheter aortic valve implantation

**DOI:** 10.1007/s10554-016-0889-x

**Published:** 2016-05-02

**Authors:** Ramón Rodríguez-Olivares, Nahid El Faquir, Zouhair Rahhab, Anne-Marie Maugenest, Nicolas M. Van Mieghem, Carl Schultz, Guenter Lauritsch, Peter P. T. de Jaegere

**Affiliations:** Department of Cardiology, Thorax Center, Erasmus Medical Center, ’s-Gravendijkwaal 230, 3015 CE Rotterdam, The Netherlands; Department of Cardiology, Royal Perth Hospital Campus, School of Medicine and Pharmacology, University of Western Australia, Crawley, Australia; Siemens Healthcare GmbH, Forchheim, Germany

**Keywords:** Aortic stenosis, Transcatheter aortic valve implantation, Rotational angiography, Image quality

## Abstract

To study the determinants of image quality of rotational angiography using dedicated research prototype software for motion compensation without rapid ventricular pacing after the implantation of four commercially available catheter-based valves. Prospective observational study including 179 consecutive patients who underwent transcatheter aortic valve implantation (TAVI) with either the Medtronic CoreValve (MCS), Edward-SAPIEN Valve (ESV), Boston Sadra Lotus (BSL) or Saint-Jude Portico Valve (SJP) in whom rotational angiography (R-angio) with motion compensation 3D image reconstruction was performed. Image quality was evaluated from grade 1 (excellent image quality) to grade 5 (strongly degraded). Distinction was made between good (grades 1, 2) and poor image quality (grades 3–5). Clinical (gender, body mass index, Agatston score, heart rate and rhythm, artifacts), procedural (valve type) and technical variables (isocentricity) were related with the image quality assessment. Image quality was good in 128 (72 %) and poor in 51 (28 %) patients. By univariable analysis only valve type (BSL) and the presence of an artefact negatively affected image quality. By multivariate analysis (in which BMI was forced into the model) BSL valve (Odds 3.5, 95 % CI [1.3–9.6], p = 0.02), presence of an artifact (Odds 2.5, 95 % CI [1.2–5.4], p = 0.02) and BMI (Odds 1.1, 95 % CI [1.0–1.2], p = 0.04) were independent predictors of poor image quality. Rotational angiography with motion compensation 3D image reconstruction using a dedicated research prototype software offers good image quality for the evaluation of frame geometry after TAVI in the majority of patients. Valve type, presence of artifacts and higher BMI negatively affect image quality.

## Introduction

Transcatheter aortic valve implantation (TAVI) is an established therapeutic option for patients with aortic stenosis who are considered at high risk or inappropriate for surgical valve replacement [1–5]. Parallel with the increasing clinical experience with various catheter-based valve technologies, there is ongoing technical improvement in these devices to overcome their technical limitations [6]. One of these is the occurrence of paravalvular aortic regurgitation that stem from a combination of patient- and procedure related variables (e.g. amount and distribution of aortic root calcium, sizing and depth of implantation) but also from intrinsic device related factors and ensuing device–host interaction that may lead to incomplete or non-uniform frame expansion [7–11]. This has led to an increased interest in on-line assessment of frame geometry to better understand and/or to improve the immediate results of TAVI [12]. Echocardiography helps to evaluate and understand valve performance but is limited in offering detailed information of the geometry of the valve frame. The latter implies (on-line) 3D imaging. Although 3D echocardiography is increasingly used to assess complex cardiac structures it is limited by sufficient temporal and spatial resolution for detailed assessment of frame geometry after TAVI. For that reason, we used rotational angiography (R-angio) using prototype software for motion compensation [12]. Conventional R-angio is characterized by a high spatial but low temporal resolution. Motion compensation techniques in the reconstruction step are restoring the good temporal resolution of the frame acquisition. R-angio, however, has been shown to be instrumental in the on-line assessment and quantification of the base of the aortic root in patients scheduled for TAVI although image quality may vary depending on a number of patient-related factors [13–15]. In this study we sought to explore the determinants of image quality of the frame geometry using R-angio with dedicated research prototype software for motion compensation without rapid ventricular pacing after the implantation of four commercially available catheter-based valves as R-angio may be used for clinical decision making of additional treatment measures, thereby, improving outcome [14, 15].

## Methods

### Patients

The study population consists of 179 consecutive patients with aortic stenosis who underwent TAVI with either the Medtronic CoreValve (MCS), Edward-SAPIEN Valve (ESV-XT & S3), Boston Sadra Lotus (BSL) or Saint-Jude Portico Valve (SJP) in whom R-angio using dedicated research prototype software for motion compensation but without rapid pacing was performed [12, 16].

R-angio was performed immediately after TAVI using the Artis zee biplane angiographic C-arm system (Siemens Healthcare GmbH, Forchheim, Germany) with a 20 × 20 cm detector and isotropic pixel length of 180 μm. A total of 133 images were acquired in 5 s along a 198° arc (99° right anterior oblique to 99° left anterior oblique view) during breath hold at a detector entrance dose of 0.36 μGy per frame and a tube voltage of 90 kV.

**Fig. 1:**
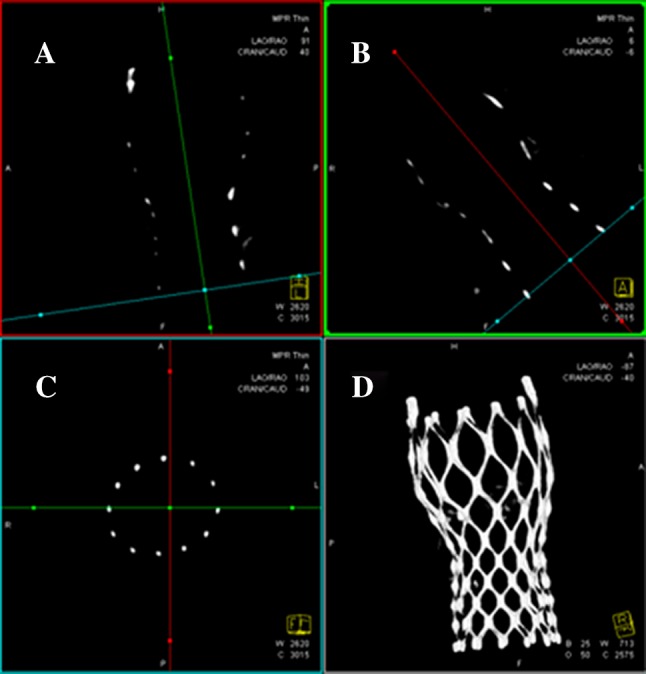
Acquisition of the multi-planar reformat short-axis view (**c**) at the different levels of interest adjusting two longitudinal multi-planer reformatted orthogonal views (**a, b**) similar to MSCT and the resulting volume rendered tridimensional reconstruction (**d**). Reprinted from EuroIntervention 2015 Aug 13;11(4) Ahead-of-print, Rodríguez-Olivares R, El Faquir N, Rahhab Z, Geeve P, Maugenest AM, van Weenen S, Ren B, Galema T, Geleijnse M, Van Mieghem NM, van Domburg R, Bruining N, Schultz C, Lauritsch G, de Jaegere PP. Does frame geometry play a role in aortic regurgitation after Medtronic CoreValve implantation? Copyright (2015), with permission from Europa Digital & Publishing

*3D reconstruction* was done using a predefined Standard Operating Procedure (supplement). From the projection data a motion compensated 3D image (R-angio) was reconstructed with research prototype software (Siemens Healthcare GmbH, Forchheim, Germany) with a matrix of 256 in each direction and isotropic (0.5 mm)^3^ voxel size. A motion compensated image reconstruction was made using the end-diastolic phase at 75 % of the cardiac cycle since at that moment there is theoretically less motion. The 3D reconstruction of the frame was then processed (e.g. cropping) before analysis. Cross-sectional short axes images were used for frame analysis (Fig. 1c).

**Fig. 2:**
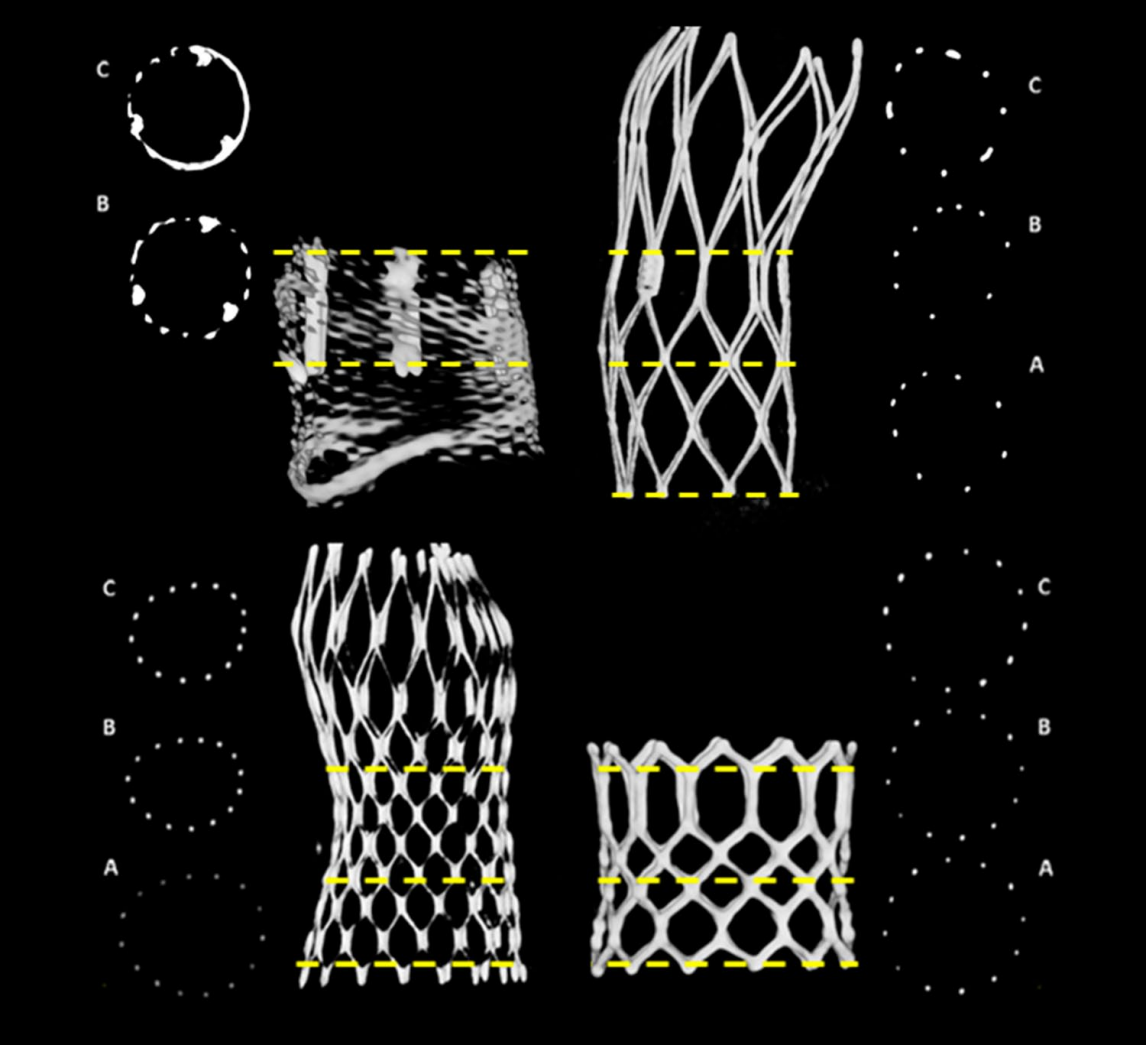
Cross-sectional view at the three levels of interest of the valve types BSL (*top left*), SJP (*top right*), MCS (*bottom left*) and ESV (*bottom right*) included in the study

Frame analysis was performed at three pre-specified levels in MCS, ESV and SJP (A—inflow, B—functioning segment and C—outflow) and in two in the BSL (functioning segment and outflow) due to the lack of useful landmarks at the level of the BSL inflow (Fig. 2).

**Fig. 3:**
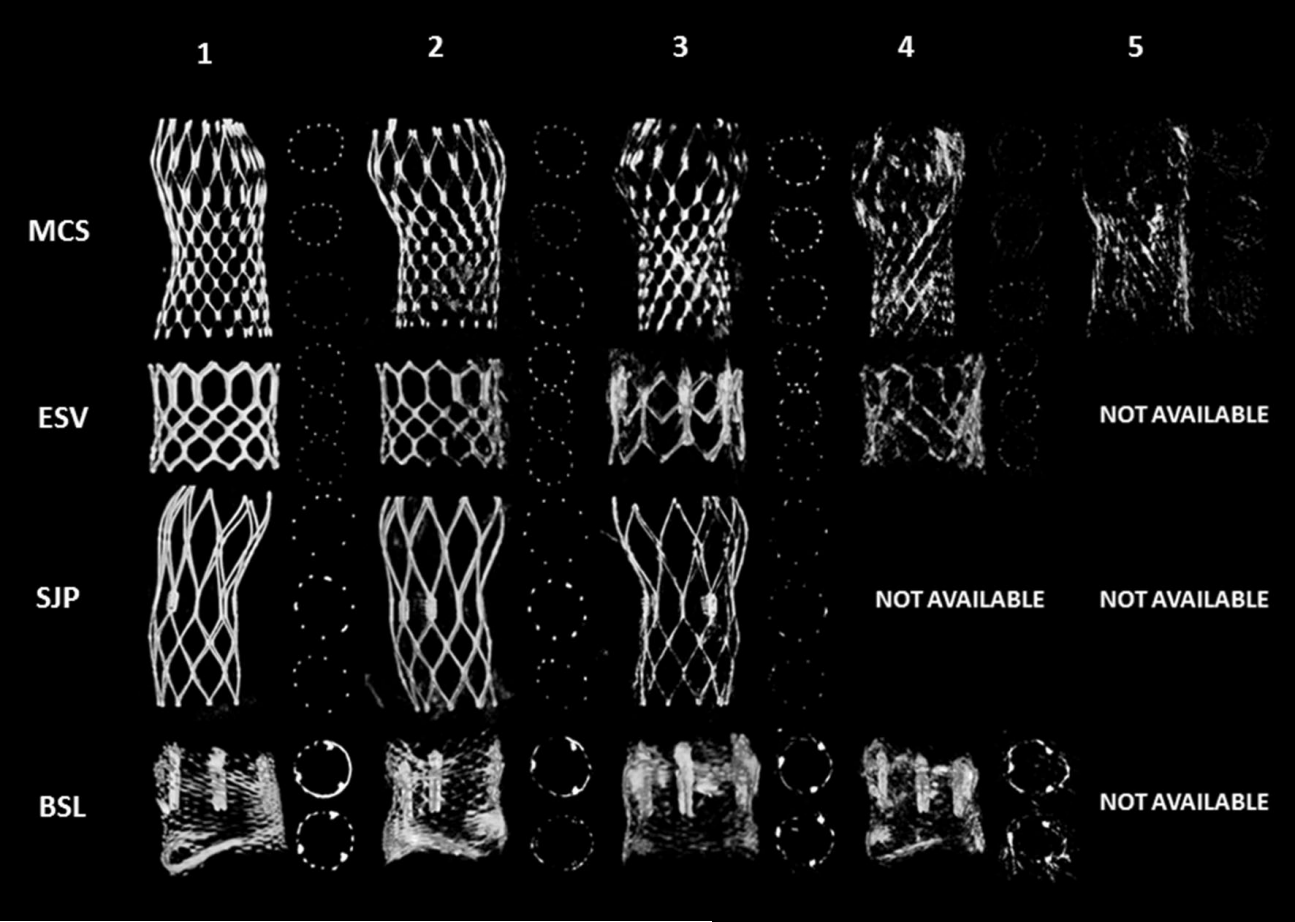
Image quality of R-angio; grade 1 (best quality) to grade 5 (worst)

Image quality was evaluated using the following score: grade 1, excellent image quality (*struts visible without artifacts*); grade 2, struts clearly visible, distinction between struts and artifacts possible; grade 3, struts visible but in some regions distinction between struts and artifacts cannot be made; grade 4, degraded (*struts are blurred and distorted*); grade 5, strongly degraded (*struts and artefacts cannot be distinguished*) (Fig. 3).

**Fig. 4:**
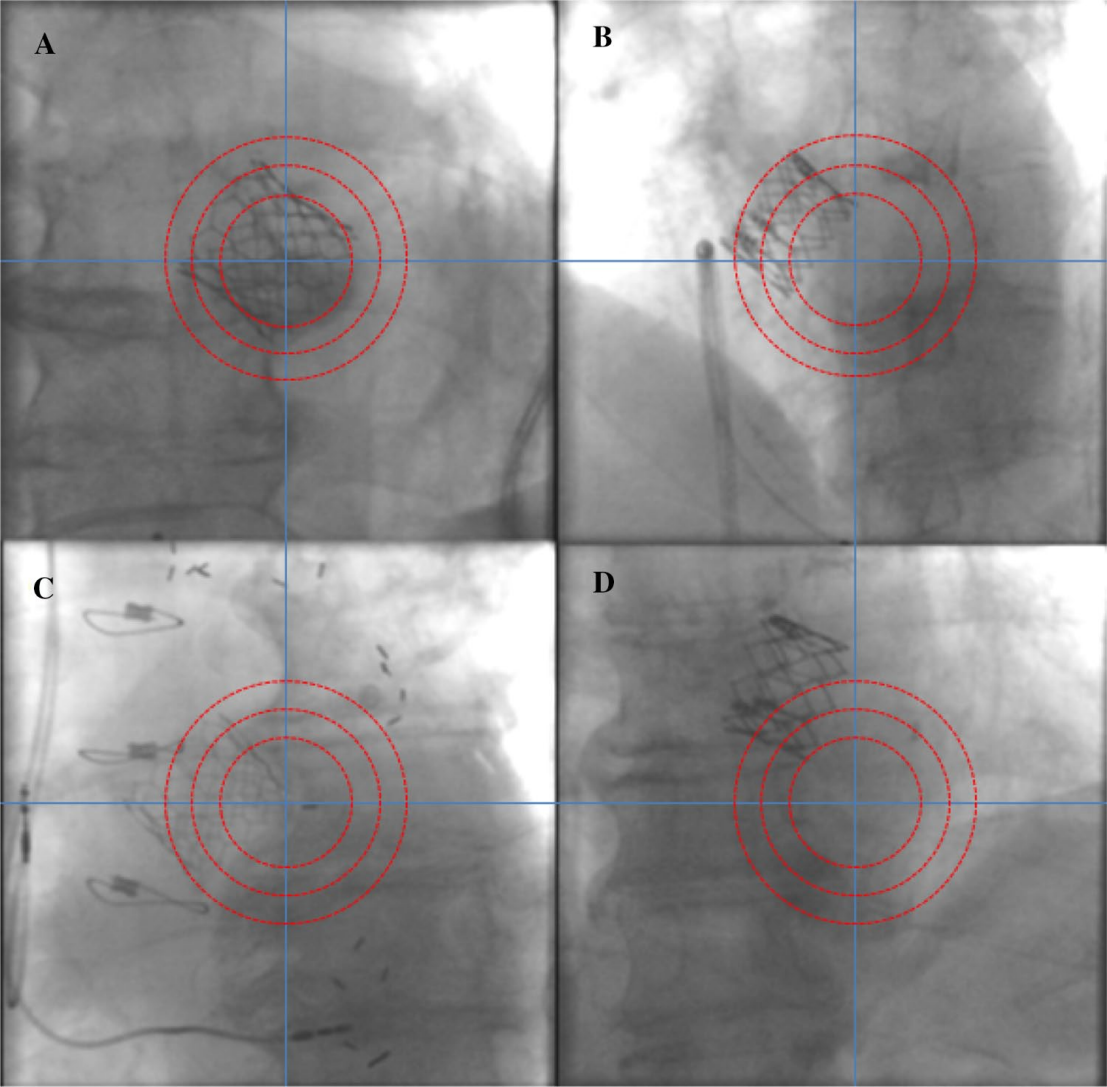
Isocentricity assessment in an implanted ESV. **a** Grade 1: “isocentric”, **b** grade 2: “slightly eccentric”, **c** grade 3: “moderately eccentric”, **d** grade 4: “severely eccentric”

Isocentricity of the valve in the field of examination was defined by drawing two orthogonal lines (vertical, horizontal) in the middle of the image window. Isocentricity was visually assessed using a circular grid consisting of three isocentric circles of different diameters and graded as isocentric (grade 1) when the valve was within the first circle, mildly off-center (grade 2) when the valve was within the boundaries of the second circle, moderately off-center when the valve was within the boundaries of the third circle and severely off-center (grade 4) when the valve was beyond the boundaries of the third circle (Fig. 4). Valve position was defined isocentric when the valve was within the boundaries of the second circle (grades 1 and 2).

**Fig. 5:**
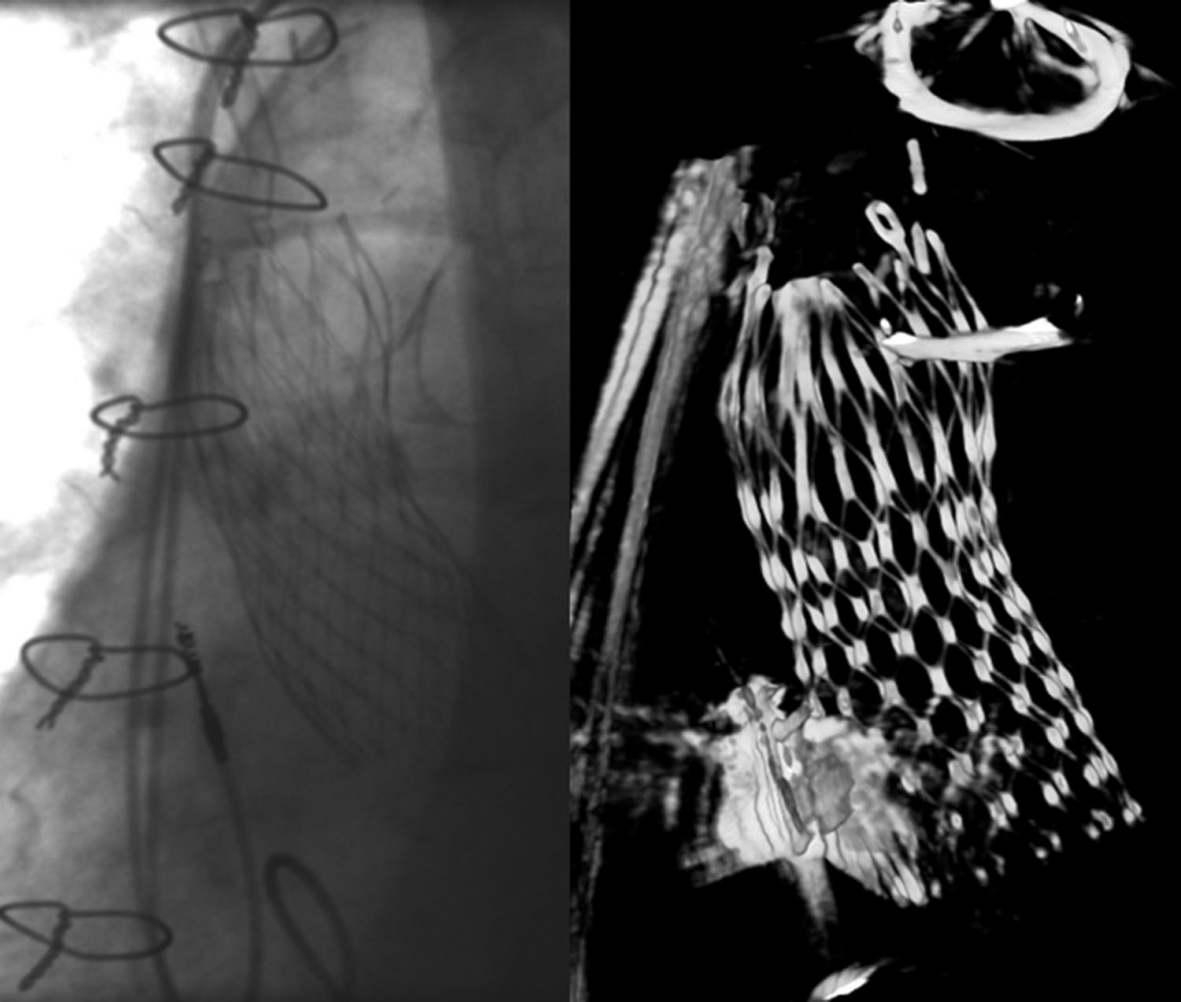
Example of the impact of artefacts (sternal wires and permanent pacemaker) on the image quality of an implanted MCS. At the left angiography, at the right 3D reconstruction using R-angio with motion compensation

An artifact was defined by the presence of at least one of the following: permanent pacemaker, transoesophageal probe, pigtail, stitches, prosthesis or other radiopaque objects in the field of examination (Fig. 5).

### Data collection, statistics and analysis

All patients gave written informed consent for the TAVI procedure as well as for anonymized analysis of all prospectively collected baseline and procedural related data (TAVI Care & Cure project, MEC-2014-277). Categorical variables are presented as frequencies and percentages and compared with the Pearson Chi square test. Continuous variables results are presented as means (±SD) and compared with the Student *t* test. To study the independent predictors of image quality multivariable logistic regression model was performed, taking into account the observed frequency of the dependent variable by (n/10). A two-sided alpha level of 0.05 was used to indicate significance.

## Results

A total of 179 patients underwent R-angio with motion compensation after TAVI using four CE marked valves (MCS, n = 98 or 54.7 %; ESV, n = 52 or 29.1 % of whom 27 XT and 25 S3; BSL, n = 23 or 12.8 %; SPJ, n = 6 or 3.4 %). The median [IQR] dose of radiation used to perform R-angio was 1351 [997–1578] cGy cm^2^ (area dose) and 183 [139–210] mGy (skin dose).

Good image quality (grade 1 or 2) was obtained in 128 patients (72 %) and poor image quality in 51 (28 %).

Per device, the prevalence of good image quality—in descending order of frequency—was ESV (45/52, 86 %), SJP (5/6, 83 %), MCS (68/98, 69 %) and BSL (10/23, 48 %). Image quality was good in 79 % of the patients when no artefact was present vs 63 % in the presence of an artifact. Arrhythmia (e.g. atrial fibrillation, premature ventricular contractions) was present in 35/179 patients (20 %) but did not affect image quality (good image quality in 29/35, 83 %) (Table 1).

**Table 1:** Baseline clinical and procedural characteristics

	Total population (n = 179)	Good image quality (n = 128)	Poor image quality (n = 51)	p value
Age (years)	79.2 ± 8.8	78.8 ± 9.0	80.3 ± 8.2	0.31
Gender, male N (%)	95 (53.1)	72 (56.2)	23 (45.1)	0.18
Height (cm)	168.6 ± 9.4	168.5 ± 9.4	168.9 ± 9.5	0.81
Weight (kg)	76.0 ± 15.4	75.0 ± 14.8	78.4 ± 16.7	0.18
Body mass index (kg/m^2^)	26.7 ± 5.0	26.4 ± 4.9	27.4 ± 5.4	0.21
Body surface area	1.9 ± 0.2	1.8 ± 0.2	1.9 ± 0.2	0.13
Agatston score	3384 ± 2231	3584 ± 2466	2892 ± 1408	0.08
Permanent pacemaker N (%)	11 (6.1)	6 (4.7)	5 (9.8)	0.20
Valve type N (%)				0.003
Corevalve	98 (54.7)	68 (53.1)	30 (54.7)	0.49
Edwards-SAPIEN	52 (29.1)	45 (35.2)	7 (13.7)	0.004
Edwards-SAPIEN XT	27 (15.1)	22 (17.2)	5 (9.8)	0.21
Edwards-SAPIEN 3	25 (14.0)	23 (18.0)	2 (3.9)	0.014
Portico	6 (3.4)	5 (3.9)	1 (2.0)	0.51
Lotus	23 (12.8)	10 (7.8)	13 (25.5)	0.001
Rhytmic N (%)	141 (78.8)	98 (77.2)	43 (87.8)	0.12
Sinus rhythm N (%)	98 (55.7)	68 (53.5)	30 (61.2)	
Pacemaker rhythm N (%)	38 (21.6)	25 (19.7)	13 (26.5)	
Other N (%)	5 (2.8)	5 (3.9)	0 (0.0)	
Arrhythmia N (%)	35 (19.9)	29 (22.8)	6 (12.2)	0.12
Sinus, nodal or pacemaker rhythm + extrasystoles N (%)	13 (7.4)	11 (8.7)	11 (4.1)	
Atrial fibrillation or atrial flutter N (%)	17 (9.7)	14 (11.0)	3 (6.1)	
Other causes of arrhythmia N (%)	5 (2.8)	4 (3.1)	1 (2.0)	
Heart rate (bpm)	75.4 ± 27.7	75.4 ± 27.8	75.3 ± 27.8	0.98
Isocentricity (≥grade 3) N (%)	59 (33.5)	41 (32.3)	18 (36.7)	0.58
Presence of any artifact N (%)	84 (47.2)	53 (41.7)	31 (60.8)	0.021

**Fig. 6:**
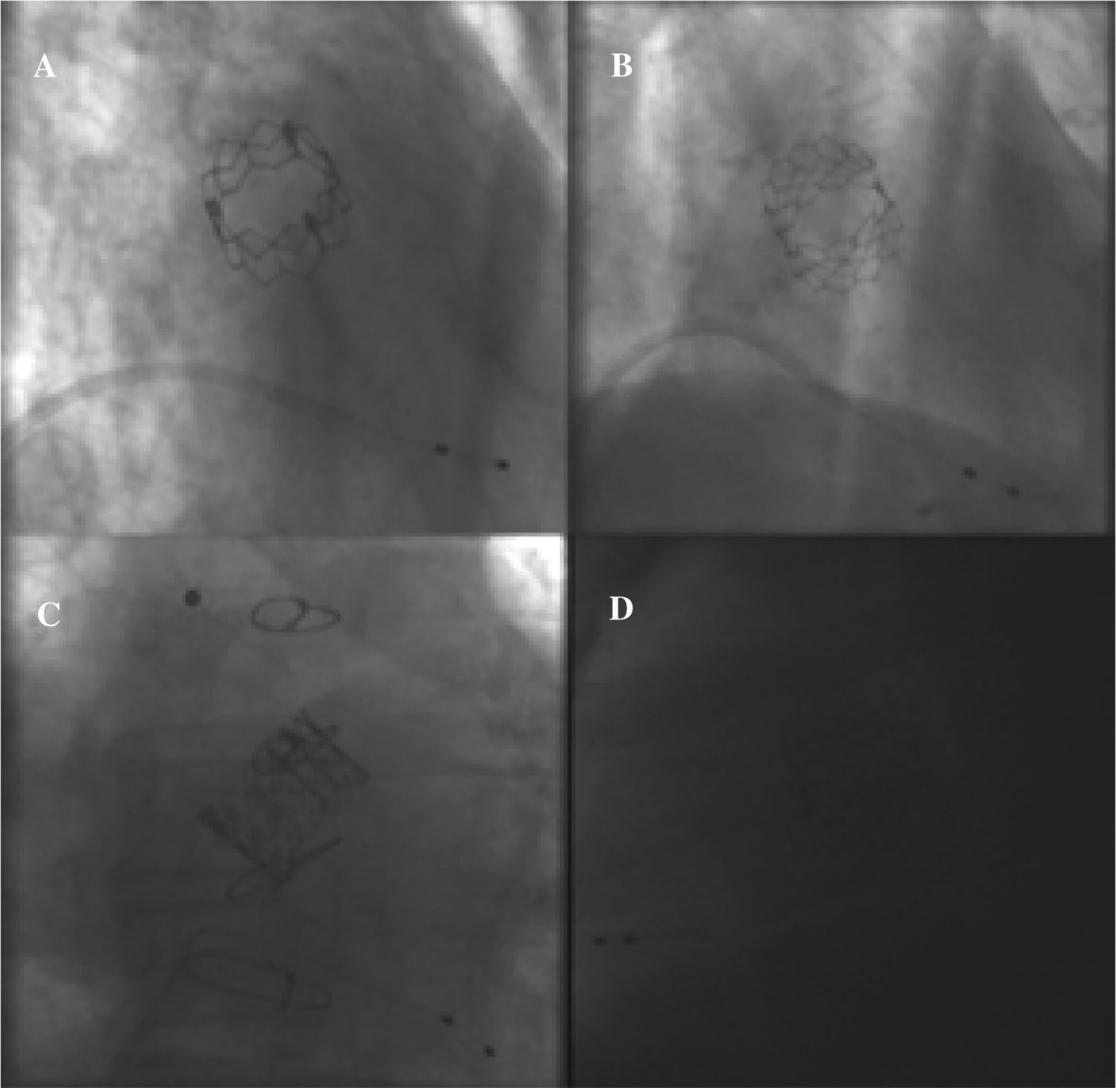
Impact of BMI on image quality. **a** BMI <20.0 (18.7), **b** BMI 20–30 (24.7), **c** BMI 30–40 (34.7), **d** BMI >40 (46.4)

By univariable analysis only valve type (BSL) and the presence of an artefact negatively affected image quality (Fig. 5). ESV was significantly related with good image quality (Table 1). By multivariate analysis (in which BMI was forced—BMI of patients with good and poor image quality: 26 ± 5 and 27 ± 5, respectively, Fig. 6), BSL valve (Odds 3.2, 95 % CI [1.2–9.0], p = 0.024), presence of an artifact (Odds 2.6, 95 % CI [1.2–4.2], p = 0.014) and BMI (Odds 1.1, 95 % CI [1.0–1.2], p = 0.034) were found to be independent predictors of poor image quality (Table 2).

**Table 2:** Univariate and multivariate logistic regression analysis

	Univariate Odds ratio (95 % CI)	p value	Multivariate Odds ratio (95 % CI)	p value
Body mass index (kg/m^2^)	1.041 (0.977–1.109)	0.21	1.081 (1.006–1.162)	0.034
Lotus	4.037 (1.638–9.947)	0.002	3.237 (1.169–8.964)	0.024
Edwards-SAPIEN	0.293 (0.122–0.705)	0.006	0.392 (0.148–1.038)	0.060
Artifacts	2.164 (1.115–4.202)	0.023	2.589 (1.216–5.511)	0.014
Isocentricity ≥grade 3	1.218 (0.611–2.428)	0.58	0.972 (0.460–2.054)	0.942

## Discussion

This study demonstrates that rotational angiography using dedicated research prototype software for motion compensation offers good image quality for the evaluation of frame geometry after TAVI in the majority of patients. Image quality is affected by patient- and procedural factors (i.e. BMI, valve type and the presence of an artifact in the field of examination). This implies that on one hand image quality will remain insufficient in a subset of patients (i.e. obese and/or receiving a Lotus valve) and, on the other, that image quality can be improved by careful execution of R-angio (e.g. avoiding artefacts).

The findings of this study must be interpreted in the light of the size and demographics of the current study population (Table 1) and the fact that R-angio was performed without rapid pacing but with prototype software for motion compensation. Similar to Schultz et al., who used R-angio for quantification of the base of the aortic root before valve implantation, we found that BMI—that was forced into the multivariate model—had a negative effect on image quality (i.e. for every increase of 1 kg, 8 % increase in risk of poor image quality) [15]. Yet, he also found that patients with a BMI <29 kg/m^2^ had good image quality regardless of rapid pacing but that good image quality was achieved in a substantial larger proportion of patients with a BMI ≥29 kg/m^2^ when image acquisition was performed when using rapid pacing (increase from 11 to 50 %) [15]. The question, therefore, is whether and to what extent rapid pacing would have contributed to improved image quality in the current study (i.e. frame analysis immediately after TAVI) and population. Rapid pacing is, however, not without harm and is preferentially to be avoided, in particular in patients whose hearts have been exposed to a long period of increased afterload [16]. It also increases the complexity and, therefore, the application of R-angio in clinical practice. X-ray settings of image acquisition are reported in the “Methods” section. A modification of the settings to increase energy output does not appear to be an option since it would increase radiation exposure to both patient and people in the immediate environment. It remains to be seen whether further improvement in software for motion compensation may improve image quality and output, potentially also in obese patients. Imaging obese patients is suffering by strong noise and poor contrast overlaid by image artifacts. These are impacting both in the accuracy of motion compensation and in degradations of the finally reconstructed images. Motion compensation might be facilitated by incorporating the information of the valve structure to be imaged, and therefore tracking a known structure. The main cause of severe image artifacts is irradiation of metallic objects like pacing electrodes. Known methods of artifact reduction from static metallic objects might be extended to moving ones. The dynamic location of the metallic object can be determined from a preliminary use of the motion compensation prototype software. Noise and remaining artifact patterns might be scaled down by nonlinear filters in the image reconstruction step.

In addition to BMI we found—not unexpectedly—that valve type played an important role in image quality. ESV proved to be a determinant of good and BSL of poor image quality. This is explained by the fact that an object with a sparse wire strut configuration (e.g. ESV) can be imaged better by motion compensation techniques than objects with a dense wire strut configuration (e.g. BSL) and—in the valves used in this study—is independent of the chemical composition of the frame that determines radio-opacity. The ESV is composed of cobalt and chromium and the BSL of nitinol that consist of nickel and titanium. All those four chemicals belong to the fourth period of transition metals and, thus, have a similar electron configuration. Irrespective of this, they have a similar atomic number or mass (ranging from 22 [titanium] to 28 [nickel] with in between chromium [27] and cobalt [27]). Given their electron configuration and mass, one may assume a similar X-ray absorption and, thus, attenuation or visibility on X-ray.

A disturbing factor is the presence of artifacts of which some can be avoided and others not. Therefore, all those measures that can improve image quality by avoiding the first category of artefacts such as the presence of a trans esophageal echo probe and catheters (pigtail in particular) should be taken care of. This also holds for putting the valve in the center of the field of examination. This is easily achieved by aligning the table and X-ray tube so that the valve is in the center of the field in the anterior–posterior projection (horizontal movement and final position of table) followed by the lateral projection (vertical movement and final position of table) without any further table movements.

**Fig. 7:**
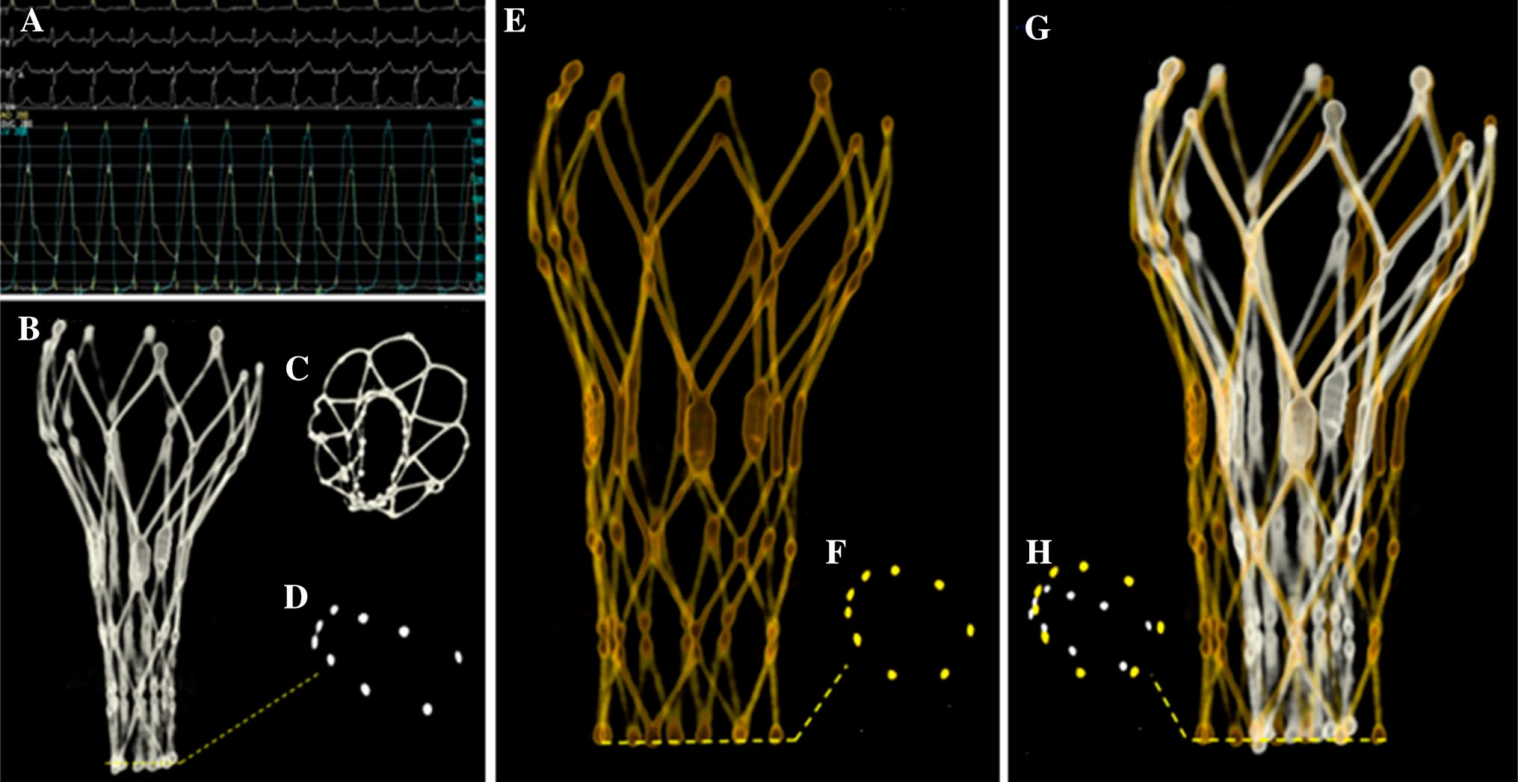
**a** Residual gradient of 68 mmHg after valve implantation. **b**–**d** Severe under-expansion of frame. **e, f** Frame geometry and dimension after balloon dilatation. **g, h** Composite of frame geometry before and after balloon dilatation

Image acquisition by R-angio and processing is on-line available in the catheterization laboratory. Image acquisition takes 5 s, no contrast is needed for frame analysis and processing of the images takes about 5 min. The combined used of the geometric analysis of the frame (R-angio) and functional analysis of the valve (echocardiography) during TAVI may offer a better understanding of the etiology of immediate valve failure or dysfunction. This is illustrated by a patient with severe paravalvular aortic regurgitation due to an unexpected high degree of frame under expansion on the basis of which balloon size was decided for additional balloon dilatation [17]. Under expansion does not per se lead to paravalvular aortic regurgitation. It may also be associated with a significant residual gradient more than regurgitation (Fig. 7). Also, R-angio may be used for on-line quantification of the aortic annulus just before TAVI and for the assessment of the aortic valve plane facilitating and improving the outcome of TAVI when MSCT is not available or not performed [15].

The relation of frame geometry assessment by R-angio and severity of aortic regurgitation (AR), have been shown in two other studies by our group [18, 19]. In patients who underwent TAVI with the MCS valve, we found a higher incidence of more-than-mild AR when the frame is more elliptical than native annulus [18]. Yet, we also found that eccentricity of the MCS frame is not always related with significant AR and that circularity of ESV frame precludes the presence of significant AR [19].

The major issue of performing R-angio is the exposure to extra radiation for patients and catheterization laboratory workers. Patients undergoing TAVI are elderly with a limited life expectancy, therefore the clinical risk associated with this exposure is negligible. During the realization of the R-angio, all workers involved in the procedure are asked to stay away from the c-arm during the image acquisition in order to minimalize radiation exposure. The operator uses the X-ray pedal at the maximum distance possible away of the c-arm taking into consideration the inverse-square law (X-ray intensity is inversely proportional to the square of the distance from the source).

## Conclusion

Rotational angiography with motion compensation 3D image reconstruction using a dedicated research prototype software offers good image quality for the evaluation of frame geometry after TAVI in the majority of patients. Valve type, presence of artifacts and higher BMI negatively affect image quality.
